# A Polyvinyl Alcohol–Tannic Acid Gel with Exceptional Mechanical Properties and Ultraviolet Resistance

**DOI:** 10.3390/gels8110751

**Published:** 2022-11-20

**Authors:** Chunqing Si, Xintong Tian, Yan Wang, Zhigang Wang, Xinfang Wang, Dongjun Lv, Aili Wang, Fang Wang, Longlong Geng, Jing Zhao, Ruofei Hu, Qingzeng Zhu

**Affiliations:** 1 College of Chemistry and Chemical Engineering, Dezhou University, Dezhou 253023, China; 2 School of Chemistry and Chemical Engineering, Shandong University, Jinan 250100, China

**Keywords:** gel, mechanical properties, stress, strain, UV-vis spectroscopy

## Abstract

Design and preparation of gels with excellent mechanical properties has garnered wide interest at present. In this paper, preparation of polyvinyl alcohol (PVA)–tannic acid (TA) gels with exceptional properties is documented. The crystallization zone and hydrogen bonding acted as physical crosslinkages fabricated by a combination of freeze–thaw treatment and a tannic acid compound. The effect of tannic acid on mechanical properties of prepared PVA–TA gels was investigated and analyzed. When the mass fraction of PVA was 20.0 wt% and soaking time was 12 h in tannic acid aqueous solution, tensile strength and the elongation at break of PVA–TA gel reached 5.97 MPa and 1450%, respectively. This PVA–TA gel was far superior to a pure 20.0 wt% PVA hydrogel treated only with the freeze–thaw process, as well as most previously reported PVA–TA gels. The toughness of a PVA–TA gel is about 14 times that of a pure PVA gel. In addition, transparent PVA–TA gels can effectively prevent ultraviolet-light-induced degradation. This study provides a novel strategy and reference for design and preparation of high-performance gels that are promising for practical application.

## 1. Introduction

Due to outstanding biofunctionality, biocompatibility, and applications in biomedicine and wearable devices, gels have been extensively explored and have attracted continuous interest [1,2,3,4]. However, intrinsic brittleness and nonideal mechanical properties of gels limit their utilization [5,6,7]. Robust mechanical properties, including high strength, stretchability, and toughness, are critical for meeting practical demands [8,9,10]. To overcome the challenges above, many efforts have been devoted to improving mechanical properties of gel through various strategies [11,12,13,14]. Mechanical strength and toughness of gel can be enhanced by block copolymerization, construction of interpenetration, hybrids, and double networks [15,16,17,18], as well as multiple interactions (such as electrostatic complex) to increase crosslinking points [19]. Mechanical properties are enhanced because of multiple “sacrificial domains” for mechanical-energy dissipation based on the dissipation-induced toughening theory [20,21]. Despite improvement in mechanical properties, these changes could lead to a significant decrease in water content and stretchability [22]. Meanwhile, any residual monomers or initiators are toxic, which is not desirable for biomedicine or wearable devices. Therefore, it has been very challenging to develop gels with a combination of superior mechanical strength and stretchability [23,24].

Tannic acid is a natural polyphenol compound. Tannic acid with catechol groups can easily form hydrogen bonds and crosslink domains with other active groups [1,25,26]. Nevertheless, because tannic acid possesses strong free-radical scavenging ability, its free-radical polymerization is inhibited and retarded [1]. Therefore, using tannic acid as a crosslinker in free-radical polymerization is difficult. Polyvinyl alcohol (PVA), thanks to its low cost, nontoxicity, and good biocompatibility, is a widely used polymeric material and has attracted extensive interest [27,28,29,30,31]. In the process of preparation of PVA, a repeated freeze–thaw process has been applied to physically crosslink via formation of a crystallization zone between PVA molecular chains [32,33,34]. However, mechanical strength and toughness of PVA hydrogels obtained only by the freeze–thaw cycle was low. Strength and toughness of PVA can be further improved through creation of abundant hydrogen-bond crosslinking points [23,35]. Introducing hydrogen-bond crosslinking points into a gel is a straightforward strategy to endow that gel with excellent mechanical properties [36,37,38]. After the freeze–thaw cycle, a PVA gel may be soaked in tannic acid to form hydrogen bonds, which would improve mechanical properties of the gel [39]. However, the processes above often require a long time of freeze-drying to obtain aerogel, and they only enhance strength, with almost no improvement in elongation [39]. As a consequence, preparation of PVA hydrogel with excellent mechanical properties remains a challenge. Moreover, due to the special structure of tannic acid, it has free-radical scavenging ability to effectively shield ultraviolet light [1,40,41]. Nevertheless, UV-radiation shielding of tannic acid has rarely been reported in previous studies of PVA hydrogels [37,39].

**Figure 1:**
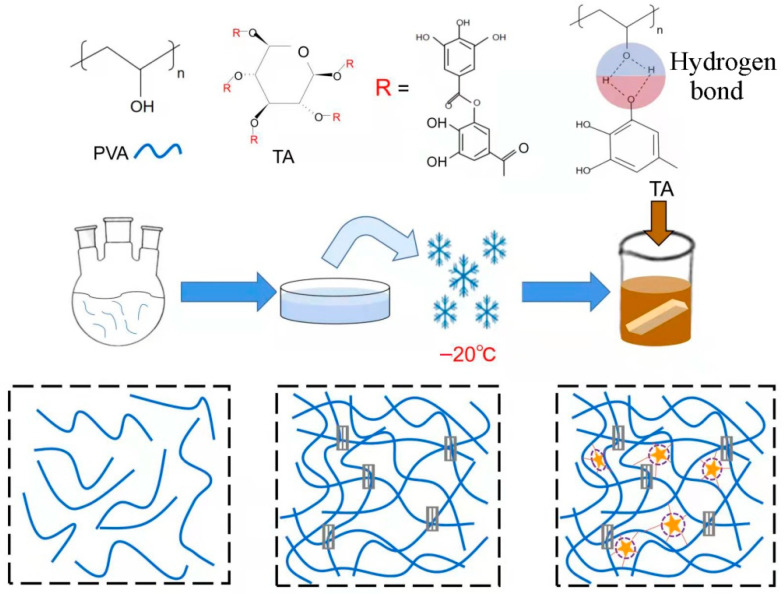
Diagram showing preparation of PVA–TA gel (third row, from left to right: PVA solution, freeze–thawed PVA gel, and PVA–TA gel).

In order to improve mechanical properties of gels, the study in this paper used PVA as a matrix gel (Figure 1). First, the physical crosslinking region was generated via the method of repeated freeze–thawing treatment. After formation of the physical crosslinking region, the surrounding void region was very conducive to the introduction of tannic acid, and hydrogen-bond crosslinks were formed, which greatly increased crosslinking points of the gel. Because of abundant and effective energy dissipation, mechanical properties of PVA–TA gel were very high. Strength and elongation at the break of the 20 wt% PVA–TA gel were 5.97 MPa and 1450%, respectively, and toughness reached 50 MJ/m^3^. In addition, tannic acid absorbed ultraviolet light so that the transparent gel was protected from ultraviolet-light-induced degradation.

## 2. Results and Discussion

Crystalline regions were formed in PVA via the freeze–thaw cycle. Simultaneously, tannic acid molecules were introduced into those crystalline regions’ surrounding space. Next, hydrogen bonds were formed between that tannic acid and PVA chains to form multiple hydrogen bonding interactions. In this way, a double crosslink, combining both crystallization regions and multiple hydrogen bonds, was formed to enhance each gel with superior mechanical properties.

**Figure 2:**
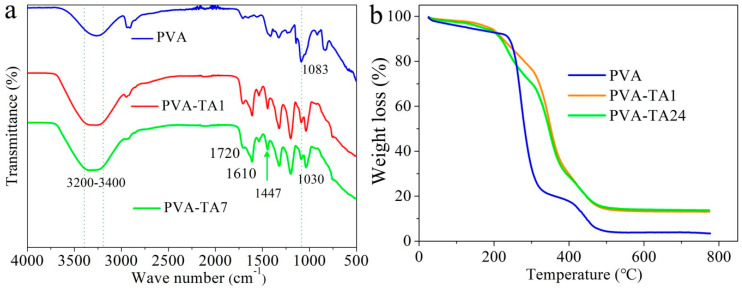
(**a**) FTIR spectra of pure PVA hydrogel and 15.0 wt% PVA–TA gel. (**b**) TG curves of pure 15.0 wt% PVA hydrogel and 15.0 wt% PVA–TA gel.

As shown in Figure 2a, for the 15.0 wt% PVA–TA gel, strong absorption peaks appeared at 3400~3200 cm^−1^ after soaking in tannic acid for 1 h and 7 h; this was caused by the hydrogen bonding association between phenolic hydroxyl groups of tannic acid and hydroxyl groups of PVA [42]. The broad and strong absorption peaks of the 15.0 wt% PVA–TA gel imply enhanced hydrogen bonding interactions as compared to those of pure PVA hydrogel [32]. FTIR spectra of tannic acid have been reported in previous studies [32,42,43]. For pure tannic acid, stretching vibrations of C=O, C=C (aromatic groups), and C-O appeared at 1730–1705 cm^−1^, at around 1450 cm^−1^, and at 1100–1300 cm^−1^. The band located at 1720 cm^−1^ could be assigned to C=O stretching vibration for the 15.0 wt% PVA–TA gel. In addition, bands at 1610 cm^−1^ and 1447 cm^−1^ could be observed in the 15.0 wt% PVA–TA gel, corresponding to the stretching vibrations of C-C in aromatic groups and the distortion vibration of C=C in benzene rings, respectively [42]. Simultaneously, for the 15.0 wt% PVA–TA gel, a new peak appeared at 1030 cm^−1^ after treatment with TA [32]. The FTIR analysis implied composition of the gel and formation of hydrogen-bond crosslinking between PVA and TA [32,42,43].

The crosslinking network could be evaluated by a thermogravimetric analyzer (TG). Thermogravimetric analysis (TGA) of tannic acid was also reported in previous studies [42,43]. Two decomposition steps—moisture loss and mass loss for tannic acid—appeared at about 38 °C and 255 °C, respectively. After 500 °C, a 28.3 wt% carbonized residue remained. Figure 2b shows influence of the crosslinking network on thermal stability of the 15.0 wt% PVA–TA gel and the pure 15.0 wt% PVA hydrogel. Decomposition of gel can be divided into two steps: water loss and mass loss. The weight loss before about 240 °C for the pure PVA hydrogel was due to evaporation of water and acetic acid in the hydrogel [44]. For the 15.0 wt% PVA–TA gel, weight loss was not obvious at about 200 °C; this was likely caused by presence of less free water than was in the pure 15.0 wt% PVA hydrogel. In the second stage, thermal decomposition of PVA occurred at about 240–500 °C for the pure 15.0 wt% PVA hydrogel. Weight loss during the second step for the 15.0 wt% PVA–TA gel owed to pyrolysis of a handful of unevaporated acetic acids, PVA molecular chains, and TA. The initial weight-loss temperature of the 15.0 wt% PVA–TA gel was lower than that of the pure PVA hydrogel in the second stage. This was presumably due to the fact that TA destroys crystallization of PVA, implying formation of interaction between PVA and TA [32]. Furthermore, the weight-loss rate of the 15.0 wt% PVA hydrogel was significantly higher than that of the 15.0 wt% PVA–TA system with an increase in temperature. For example, abrupt loss in weight started at 240 °C for the 15.0 wt% PVA hydrogel, but did not occur until about 300 °C for the 15.0 wt% PVA–TA gel, suggesting that the hydrogen bond formed between PVA and TA increases thermal stability of the gel [32]. Finally, the weight-loss curves after 1 h and 24 h soaking times were very similar, with a return to a stable weightlessness platform when the polymer almost completely decomposed to generate carbon residue.

**Figure 3:**
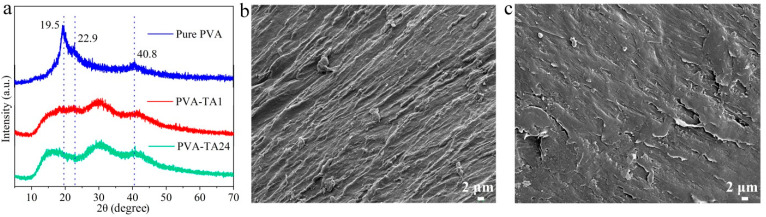
(**a**) XRD patterns of pure 15.0 wt% PVA gel and 15.0 wt% PVA–TA gel; (**b**,**c**) SEM image of 15.0 wt% PVA–TA24 gel.

X-ray diffraction (XRD) was used to investigate the structure of the pure 15.0 wt% PVA hydrogel and that of the 15.0 wt% PVA–TA24 gel. As illustrated in Figure 3a, there were three typical peaks—at 2 θ = 19.5°, 2 θ = 22.9°, and 2 θ = 40.8°—for the pure PVA hydrogel. This can be assigned to the (101), (200), and (102) planes of PVA crystallites [40,45,46]. For the 15.0 wt% PVA–TA1 gel and the 15.0 wt% PVA–TA24 gel, the XRD pattern only displayed a diffusion peak at 2 θ = 30.0°, which may have simply indicated a smaller or less orderly crystalline structure. Therein, PVA chains were restricted by the strong hydrogen bond between PVA and TA [40]. Additionally, PVA microstructure was observed using field-emission scanning electron microscopy (SEM). The 15.0 wt% PVA–TA24 gel was first freeze-dried and then cut by scissors. The fracture and surface structure of the 15.0 wt% PVA–TA24 gel can be seen in Figure 3b,c, respectively. The fracture structure was similar to fiber bundles and delamination with about a 0.2 μm thickness. The surface showed a relatively flat surface and a few layered structures.

**Figure 4:**
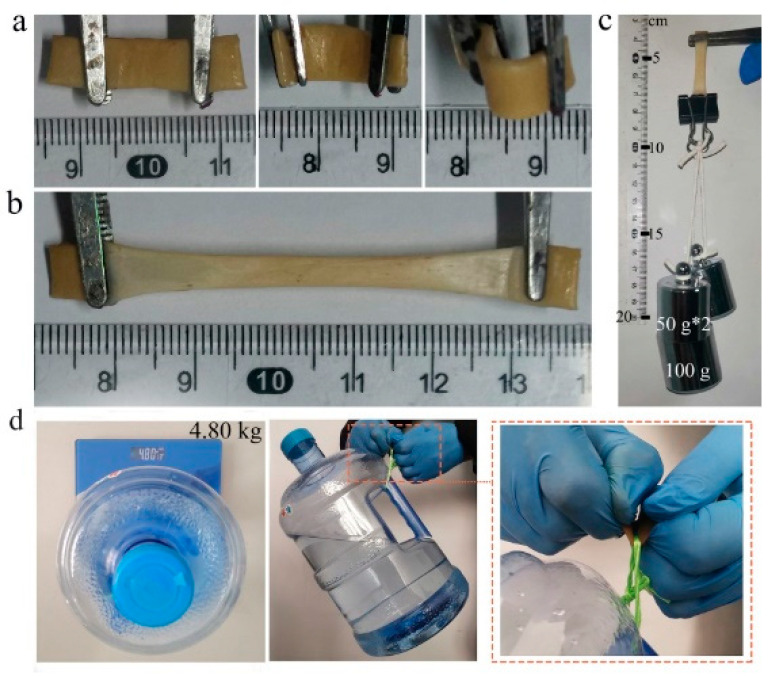
Images of 20.0 wt% PVA–TA24 gel mechanical properties: (**a**) bending, (**b**) stretching, and (**c**,**d**) holding a weight of 200 g and 4.80 kg.

The PVA–TA gel displayed excellent mechanical properties. As can be seen in Figure 4a–c, the 20.0 wt% PVA–TA24 gel could be stretched from 1 cm to about 5 cm and could easily lift a weight of 200 g. The 20.0 wt% PVA–TA24 gel (1.65 cm wide and 0.128 cm thick) could also lift a weight of 4.8 kg, as is evident in Figure 4d.

**Figure 5:**
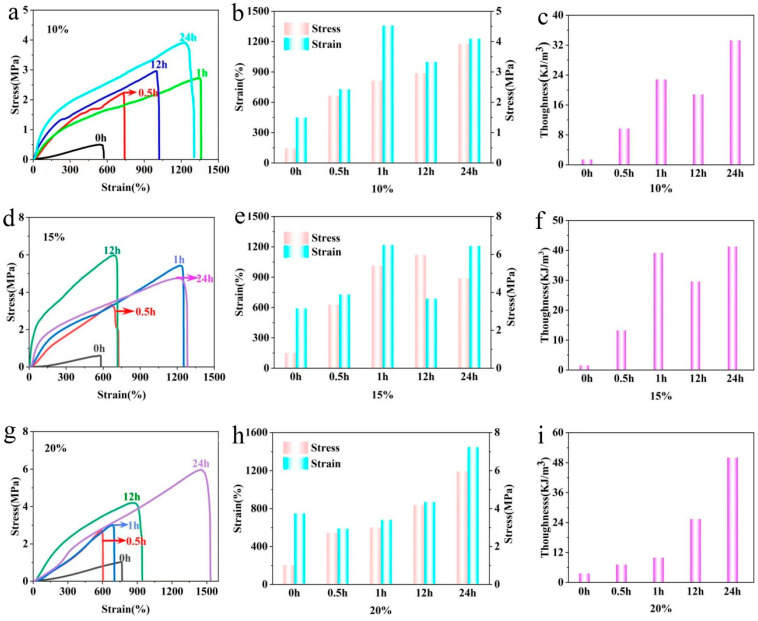
For 10.0 wt% PVA–TA gel: stress–strain curves (**a**), corresponding rupture stress–strain (**b**) and toughness values (**c**). For 15.0 wt% PVA–TA gel: stress–strain curves (**d**), corresponding rupture stress–strain (**e**) and toughness values (**f**). For 20.0 wt% PVA–TA gel: stress–strain curves (**g**), corresponding rupture stress/strain (**h**) and toughness values (**i**).

Typical tensile stress–strain curves of each gel are displayed in Figure 5a,d,g. Tensile strength, elongation at break, and toughness of each gel are summarized in Figure 5b,c,e,f,h,i, respectively. Tensile strength and elongation of the pure PVA hydrogel were lower than those of the PVA–TA gel. Strength and toughness values experienced a process of increasing, decreasing, and increasing again with extension of soaking time, according to the curves for the 10% and 15% samples, while the 20% samples showed a monotonic increase. The reason may have been that a short immersion time leads to too few tannic acid and hydrogen bonds, while a long immersion time leads to tannic acid concentration equilibrium and hydrogen bond rearrangement (Table 1). When the mass fraction was high, concentration of tannic acid and the number of hydrogen bonds slowly increased due to high intermolecular density. Therefore, tensile strength and elongation at break of the 20.0 wt% PVA–TA gel gradually increased with time. At 24 h, the 20% wt% PVA–TA24 gel had the highest strength, elongation, and toughness. The 20.0 wt% PVA–TA gel evidenced high toughness, of 50 MJ m^−3^, with an ultrahigh ultimate stress value of 5.97 MPa and ultimate strain of 1450% (Figure 5g). The toughness of the 20.0 wt% PVA–TA gel after soaking in tannic acid for 24 h was about 14 times that of the pure 20.0 wt% PVA hydrogel, which was better than reported in the previous literature (Table 2).

**Figure 6:**
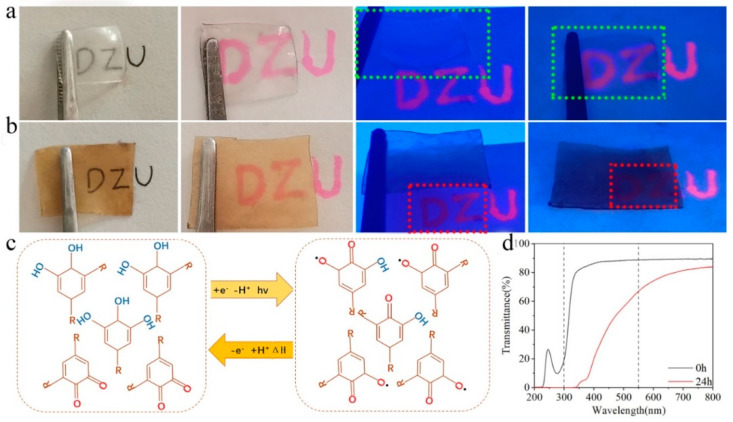
UV-radiation shielding of 15.0 wt% PVA–TA gel: (**a**) pure PVA gel under natural light (two images on the left) and ultraviolet light (two images on the right; wavelength of ultraviolet lamps was 365 nm). (**b**) Samples of 15.0 wt% PVA–TA24 gel (thickness: 0.12 cm) under natural light (two images on the left) and ultraviolet light (two images on the right). (**c**) Schematics illustrating the UV filtration process of gel derived from TA molecules. (**d**) Transmittance spectra of 15.0 wt% PVA–TA24 gel in the visible wavelength range (during 200–800 nm).

The gel effectively blocked penetration of UV rays because of the tannic acid. Herein, the 15.0 wt% PVA–TA24 gel (TA, 24 h) was selected for UV analysis. Figure 6a,b show rhodamine dye handwriting on the pure PVA hydrogel and on the 15.0 wt% PVA–TA24 gel. The three letters “DZU” could be clearly seen, indicating that natural visible light could pass through. A UV lamp was used to test UV-light absorption or blocking capacity of the 15.0 wt% PVA–TA24 gel. The pure PVA hydrogel not soaked in tannic acid did not absorb UV light. Rhodamine writing showed a fluorescence effect under the UV lamp, whether covered by the gel or not (Figure 6a). When the 15.0 wt% PVA–TA24 gel was partially placed over the dye writing, the “DZ” letters were darkened and only the “U” showed a fluorescent effect, indicating that the 15.0 wt% PVA–TA24 gel blocked the UV light. Tannic acid endowed the gel with excellent UV blocking performance due to phenolic quinone reversible tautomerism and a π electron conjugation effect [48]. Figure 6c shows the mechanism for UV light absorption. A 320–400 nm spectrum possessed mostly strong penetration, and our eyes were the most sensitive to 550 nm. Therefore, 300–550 nm was chosen for further comparison and analysis. As demonstrated in Figure 6d, the pure 15.0 wt% PVA hydrogel had excellent visible light transmittance but no blocking of ultraviolet light transmission. Intriguingly, the 15.0 wt% PVA–TA24 gel not only showed transparency in the visible light region but also had a certain ability to block ultraviolet light. It could be seen that tannic acid had excellent UV-radiation shielding ability, which could effectively solve the deficiency of traditional gels in UV resistance.

## 3. Conclusions

PVA–TA gels with high elongation, high strength, high toughness, and enhanced UV-radiation shielding were synthesized. The PVA–TA gel provided high elongation at a break of 1450%, strength of 5.97 MPa, and toughness of 50 MJ m^−3^. These good mechanical properties were due to the crystallization region formed by freeze–thaw cycles and multiple hydrogen-bond crosslinking between the tannic acid and the molecular chain. In addition, because tannic acid has phenol-quinone reversible tautomerism and a π electron conjugation effect, a PVA–TA gel can effectively absorb ultraviolet light. Because of its excellent mechanical properties and UV resistance, this gel gives a solid foundation for potential applications, such as load-bearing devices and fabrication of artificial materials. This study also provides an important reference for study and improvement of mechanical properties of gels.

## 4. Experimental Section

### 4.1. Materials

Polyvinyl alcohol (PVA, 1797, molecular weight 74,800) was purchased from Shanghai Macklin Biochemical Co. LTD. Tannic acid (purity 97.51%) and acetic acid (36%) were supplied by Tianjin Shengao Chemical Reagent Co. LTD and Tianjin Damao Chemical Reagent Factory, respectively. All chemicals were used directly without further purification. Deionized water was used for all experiments.

### 4.2. Preparation of PVA–TA Gel

PVAs with mass fractions of 10.0 wt%, 15.0 wt%, and 20.0 wt% were prepared and uniformly dissolved in water (containing 36% acetic acid; m_H2O_:m_36%acetic acid_ = 9:1) at 90 °C. PVA was then poured into a 1 mm thick polyvinylidene fluoride mold and frozen at −20 °C for 2 h. The temperature was raised to 25 °C, then cooled back down to −20 °C and held for another two hours. After three cycles of freeze–thawing, each gel was soaked in saturated tannic acid solution for 0.5 h, 1 h, 12 h, and 24 h, respectively. Finally, each gel was frozen again and then raised to room temperature to obtain PVA–TA gels (Figure 1). The control sample was prepared with a similar process, but without soaking in tannic acid solution (Table 1).

### 4.3. Characterization

Fourier transform infrared (FTIR) spectroscopy of the gel was collected by a Nicolet iS50 FT-IR spectrometer (Thermo Scientific Fisher, USA) in the wavelength range of 500–4000 cm^−1^. The microstructure of the gel was analyzed by field emission scanning electron microscopy (SEM, Merlin Compact, Zeiss, Germany). Specimens were freeze-dried in vacuum and then sputtered with gold for 30 s. Crystallinity was investigated on an X-ray diffractometer (XRD, D8 Advance, Bruker, Germany) with Cu Kα radiation (sample–detector distance was about 21 cm). Mechanical property tests were performed under ambient conditions, using a universal testing machine (Jinan Marxtest Technology Co., Ltd., Jinan, China) with a 50 N load-cell (precision was 0.0002 N). Each gel was cut into rectangles (20 mm × 5 mm × 1 mm), and a tensile test of each gel with a 10 mm initial distance was carried out at a rate of 100 mm/min. The tensile test was carried out three times for each type of gel. To obtain the light-filtering capability measurement, the fluorescent mark “DZU” was irradiated by UV lamps (364 nm), then covered with the pure PVA hydrogel and the 15.0 wt% PVA–TA gel, respectively. Resultant light was photographed with a mobile phone camera. Visible and UV transmittance of gel were collected on an UV2700 (Shimadzu Co., Japan) in the wavelength range of 800 nm to 200 nm. A thermogravimetric analyzer (Netzsch STA 449 F5 Jupiter, Germany) was used to determine thermal stability of samples with a 20 mL/min nitrogen flow rate. The temperature was raised from room temperature to 800 °C at a heating rate of 10 °C/min.

## Figures and Tables

**Table 1:** Composition of PVA–TA gel.

Time (h)	0 h	0.5 h	1 h	7 h	12 h	24 h
10.0 wt% Gel	10.0 wt% PVA	10.0 wt% PVA–TA0.5	10.0 wt% PVA–TA1	10.0 wt% PVA–TA7	10.0 wt% PVA–TA12	10.0 wt% PVA–TA24
15.0 wt% Gel	15.0 wt% PVA	15.0 wt% PVA–TA0.5	15.0 wt% PVA–TA1	15.0 wt% PVA–TA7	15.0 wt% PVA–TA12	15.0 wt% PVA–TA24
20.0 wt% Gel	20.0 wt% PVA	20.0 wt% PVA–TA0.5	20.0 wt% PVA–TA1	20.0 wt% PVA–TA7	20.0 wt% PVA–TA12	20.0 wt% PVA–TA24

**Table 2:** Comparison of mechanical properties of PVA–TA gel with those of previous PVA composite gel.

Gel	Stress MPa	Elongation at Break %	Toughness MJ/m^3^	Reference
PVA–tannic acid	9.5	1000	/	[1]
Silk sericin/PVA/sodium citrate	4.42 ± 1.05	585 ± 34.0	13.73 ± 1.05	[20]
PVA/HCPE nanocomposites	98	550	425	[23]
PVA–tannic acid@SiO_2_ hydrogel	15.7	487	/	[32]
PVA–tannic acid hydrogel	19.3	Around 300	32.1	[39]
PVA–tannic acid hydrogel	2.88	1100	/	[40]
PVA/NaCl hydrogel	1.5	550	4.7	[47]
This study	5.97	1450	50	

## Data Availability

The data that support the findings of the current study are listed within the article.

## References

[1] Fan H., Wang J., Jin Z. (2018). Tough, swelling-resistant, self-healing, and adhesive dual-cross-linked hydrogels based on polymer-tannic acid multiple hydrogen bonds. Macromolecules.

[2] Yuan Y., Zhou J., Lu G., Sun J., Tang L. (2021). Highly stretchable, transparent, and self-adhesive ionic conductor for high-performance flexible sensors. ACS Appl. Polym. Mater..

[3] Xu K., Wang Y., Zhang B., Zhang C., Liu T. (2021). Stretchable and self-healing polyvinyl alcohol/cellulose nanofiber nanocomposite hydrogels for strain sensors with high sensitivity and linearity. Compos. Commun..

[4] Lee J.N., Lee S.Y., Park W.H. (2021). Bioinspired Self-Healable Polyallylamine-Based Hydrogels for Wet Adhesion: Synergistic Contributions of Catechol-Amino Functionalities and Nanosilicate. ACS Appl. Mater. Interfaces.

[5] Zhao X., Chen X., Yu H., Lin S., Liu X., Parada G. (2021). Soft materials by design: Unconventional polymer networks give extreme properties. Chem. Rev..

[6] Mokoena T.E., Magagula S.I., Mochane M.J., Mokhena T.C. (2021). Mechanical properties, thermal conductivity, and modeling of boron nitride-based polymer composites: A review. Extreme Mech. Lett..

[7] Shejkar S.K., Agrawal B., Agrawal A., Gupta G. (2022). Physical, mechanical, and sliding wear behavior of epoxy composites filled with surface modified walnut shell particulate. Polym. Composite..

[8] Zhou Q., Lyu J., Wang G., Robertson M., Qiang Z., Sun B., Ye C., Zhu M. (2021). Mechanically strong and multifunctional hybrid hydrogels with ultrahigh electrical conductivity. Adv. Funct. Mater..

[9] Hu R., Ji G., Zhao J., Gu X., Zhou L., Zheng J. (2022). The preparation of dual cross-linked high strain composite gel with manifold excellent properties and its application as a strain sensor. Compos. Sci. Technol..

[10] Norioka C., Inamoto Y., Hajime C., Kawamura A., Miyata T. (2021). A universal method to easily design tough and stretchable hydrogels. NPG Asia Mater..

[11] Kim J., Zhang G., Shi M., Suo Z. (2021). Fracture, fatigue, and friction of polymers in which entanglements greatly outnumber cross-links. Science.

[12] Kim S., Regitsky A.U., Song J., Ilavsky J., McKinley G.H., Holten-Andersen N. (2021). In situ mechanical reinforcement of polymer hydrogels via metal-coordinated crosslink mineralization. Nat. Comm..

[13] Steck J., Kim J., Yang J., Hassan S., Suo Z. (2020). Topological adhesion. I. Rapid and strong topohesives. Extreme Mech. Lett..

[14] Vlad-Bubulac T., Hamciuc C., Rimbu C.M., Aflori M., Butnaru M., Enache A.A., Serbezeanu D. (2022). Fabrication of Poly(vinyl alcohol)/Chitosan Composite Films Strengthened with Titanium Dioxide and Polyphosphonate Additives for Packaging Applications. Gels.

[15] Gong M., Wan P. (2021). Bioinspired stiff yet tough healable nanocomposites: From molecular design to structural processing. Matter.

[16] Li M.-X., Rong M.-Z., Zhang M.-Q. (2021). Reversible mechanochemistry enabled autonomous sustaining of robustness of polymers-an example of next generation self-healing strategy. Chin. J. Polym. Sci..

[17] Zhao J., Ji G., Li Y., Hu R., Zheng J. (2021). Preparation of a self-healing polyaniline-based gel and its application as a healable all-in-one capacitor. Chem. Eng. J..

[18] Brassinne J., Zhuge F., Fustin C.-A., Gohy J.-F. (2015). Precise Control over the Rheological Behavior of Associating Stimuli-Responsive Block Copolymer Gels. Gels.

[19] Sciortino F., Mir S.H., Pakdel A., Oruganti A., Abe H., Witecka A., Awang Shri D.N., Rydzek G., Ariga K. (2020). Saloplastics as multiresponsive ion exchange reservoirs and catalyst supports. J. Mater. Chem. A.

[20] Wang F., Li Z., Guo J., Liu L., Fu H., Yao J., Krucińska I., Draczyński Z. (2021). Highly strong, tough, and stretchable conductive hydrogels based on silk sericin-mediated multiple physical interactions for flexible sensors. ACS Appl. Polym. Mater..

[21] Ouyang K., Zhuang J., Chen C., Wang X., Xu M., Xu Z. (2021). Gradient diffusion anisotropic carboxymethyl cellulose hydrogels for strain sensors. Biomacromolecules.

[22] Li Y., Li S., Sun J. (2021). Degradable poly(vinyl alcohol)-based supramolecular plastics with high mechanical strength in a watery environment. Adv. Mater..

[23] Liu L., Zhu M., Xu X., Li X., Ma Z., Jiang Z., Pich A., Wang H., Song P. (2021). Dynamic nanoconfinement enabled highly stretchable and supratough polymeric materials with desirable healability and biocompatibility. Adv. Mater..

[24] Hua M., Wu S., Ma Y., Zhao Y., Chen Z., Frenkel I., Strzalka J., Zhou H., Zhu X., He X. (2021). Strong tough hydrogels via the synergy of freeze-casting and salting out. Nature.

[25] Wang N., Feng X., Pei J., Cui Q., Li Y., Liu H., Zhang X. (2022). Biobased Reversible Cross-Linking Enables Self-Healing and Reprocessing of Epoxy Resins. ACS Sustain. Chem. Eng..

[26] Heidarian P., Gharaie S., Yousefi H., Paulino M., Kaynak A., Varley R., Kouzani A.Z. (2022). A 3D printable dynamic nanocellulose/nanochitin self-healing hydrogel and soft strain sensor. Carbohyd. Polym..

[27] Hu R., Ji G., Wang Y., Zhao J., Zheng J. (2021). Rational design of multiple hydrogen bonds to improve the mechanical property of rigid PANI. Extreme Mech. Lett..

[28] Xue S., Wu Y., Liu G., Guo M., Liu Y., Zhang T., Wang Z. (2021). Hierarchically reversible crosslinking polymeric hydrogels with highly efficient self-healing, robust mechanical properties, and double-driven shape memory behavior. J. Mater. Chem. A.

[29] Ji Y., Liang N., Xu J., Zuo D., Chen D., Zhang H. (2018). Cellulose and poly(vinyl alcohol) composite gels as separators for quasi-solid-state electric double layer capacitors. Cellulose.

[30] Oral I., Ekrem M. (2022). Measurement of the elastic properties of epoxy resin/polyvinyl alcohol nanocomposites by ultrasonic wave velocities. Extreme Mech. Lett..

[31] Jalageri M.B., Mohan Kumar G.C. (2022). Hydroxyapatite Reinforced Polyvinyl Alcohol/Polyvinyl Pyrrolidone Based Hydrogel for Cartilage Replacement. Gels.

[32] Bai Z., Jia K., Liu C., Wang L., Lin G., Huang Y., Liu S., Liu X. (2021). A solvent regulated hydrogen bond crosslinking strategy to prepare robust hydrogel paint for oil/water separation. Adv. Funct. Mater..

[33] Zhao J., Li J., Zeng Q., Wang H., Yu J., Ren K., Dai Z., Zhang H., Zheng J., Hu R. (2022). A chewing gum residue-based gel with superior mechanical properties and self-healability for flexible wearable sensor. Macromol. Rapid Commun..

[34] Liang N., Ji Y., Zuo D., Zhang H., Xu J. (2019). Improved performance of carbon-based supercapacitors with sulfonated poly(ether ether ketone)/poly(vinyl alcohol) composite membranes as separators. Polym. Int..

[35] De la Cruz L.G., Abt T., Leon N., Wang L., Sanchez-Soto M. (2022). Ice-Template Crosslinked PVA Aerogels Modified with Tannic Acid and Sodium Alginate. Gels.

[36] He X., Zeng Y., Liu G., Tian Y., Wei Y., Zhao L., Yang L., Tao L. (2022). Magnetic self-healing hydrogel from difunctional polymers prepared via the kabachnik-fields reaction. ACS Macro Lett..

[37] Hu R., Zhao J., Wang Y., Li Z., Zheng J. (2019). A highly stretchable, self-healing, recyclable and interfacial adhesion gel: Preparation, characterization and applications. Chem. Eng. J..

[38] Wang J., Liu F., Tao F., Pan Q. (2017). Rationally designed self-healing hydrogel electrolyte toward a smart and sustainable supercapacitor. ACS Appl. Mater. Interfaces.

[39] Luo C., Huang M., Sun X., Wei N., Shi H., Li H., Lin M., Sun J. (2022). Super-strong, nonswellable, and biocompatible hydrogels inspired by human tendons. ACS Appl. Mater. Interfaces.

[40] Chen Y.N., Peng L., Liu T., Wang Y., Shi S., Wang H. (2016). Poly(vinyl alcohol)-tannic acid hydrogels with excellent mechanical properties and shape memory behaviors. ACS Appl. Mater. Interfaces.

[41] Cencha L.G., Allasia M., Passeggi M.C.G., Gugliotta L.M., Minari R.J. (2021). Formulation of self-crosslinkable hybrid acrylic/casein latex by tannic acid. Prog. Org. Coat..

[42] Pantoja-Castro M.A., González-Rodríguez H. (2011). Study by infrared spectroscopy and thermogravimetric analysis of tannins and tannic acid. Rev. Latinoam. Quim..

[43] Lei H., Zhao J., Ma X., Li H., Fan D. (2021). Antibacterial dual network hydrogels for sensing and human health monitoring. Adv. Health. Mater..

[44] Li Y., Song Y., Li J., Li Y., Li N., Niu S. (2018). A scalable ultrasonic-assisted and foaming combination method preparation polyvinyl alcohol/phytic acid polymer sponge with thermal stability and conductive capability. Ultrason. Sonochem..

[45] Peng M., Xiao G., Tang X., Zhou Y. (2014). Hydrogen-bonding assembly of rigid-rod poly(p-sulfophenylene terephthalamide) and flexible-chain poly(vinyl alcohol) for transparent, strong, and tough molecular composites. Macromolecules.

[46] Bai Z., Wang T., Zheng X., Huang Y., Chen Y., Dan W. (2020). High strength and bioactivity polyvinyl alcohol/collagen composite hydrogel with tannic acid as cross-linker. Polym. Eng. Sci..

[47] Wang Q., Zhang Q., Wang G., Wang Y., Ren X., Gao G. (2022). Muscle-inspired anisotropic hydrogel strain sensors. ACS Appl. Mater. Interfaces.

[48] Wei Y., Xiang L., Zhu P., Qian Y., Zhao B., Chen G. (2021). Multifunctional organohydrogel-based ionic skin for capacitance and temperature sensing toward intelligent skin-like devices. Chem. Mater..

